# Study of Serum Gamma-Glutamyl Transferase Levels in Patients With Acute Ischemic Stroke

**DOI:** 10.7759/cureus.65336

**Published:** 2024-07-25

**Authors:** Eeshaan Rathor, Chinmayee Arasada, Ashwin Kulkarni, Mohammed Aslam Shaikh

**Affiliations:** 1 Internal Medicine, Ramaiah Medical College, Bengaluru, IND

**Keywords:** “stroke severity”, oxidative stress and free radicals, glutathione, gamma glutamyl transferase (ggt), serum gamma-glutamyl transferase, national institute of health stroke score (nihss), acute ischemic stroke (ais)

## Abstract

Background

Gamma-glutamyl transferase (GGT) mediates intracellular uptake of glutathione which is a known antioxidant. GGT levels are found to be elevated in conditions of oxidative stress. Ischemic stroke results in anoxic injury, which liberates free radicals, causing glutathione to rise, which may be accompanied by a rise in serum GGT levels. This study aimed to compare serum GGT levels in acute ischemic stroke patients with normal controls and to ascertain the relation of serum GGT levels with National Institute of Health Stroke Scale (NIHSS) scores.

Materials and methods

This cross-sectional study was carried out in a tertiary care hospital in South India from August 2023 to February 2024. The study included 57 patients who presented with acute ischemic stroke within 24 hours of onset and 57 age- and sex-matched controls. The serum GGT levels of the cases were compared with age- and sex-matched controls using an independent t-test. Mean serum GGT levels were compared among groups with varying NIHSS scores and different locations of infarction using the ANOVA test. Serum GGT levels were also compared based on age, gender, and various comorbidities.

Results

The mean serum GGT levels were significantly increased (p < 0.0001) in acute ischemic stroke patients, 43.96 ± 28.02 (mean ± SD), when compared to controls, 26.14 ± 5.93 (mean ± SD). The difference in serum GGT levels with NIHSS scores of 5-15 (moderate strokes) with 34.17 ± 18.39 (mean ± SD), 16-20 (moderate-severe strokes) with 46.64 ± 21.95 (mean ± SD), and >21 (severe stroke) with 84.62 ± 39.35 (mean ± SD) was significant (p < 0.00001). Serum GGT levels were not significant while comparing age, gender, location of infarction, type 2 diabetes mellitus, and hypertension.

Conclusion

Serum GGT levels were significantly elevated in acute ischemic stroke patients within 24 hours of presentation. Serum GGT levels were significantly elevated with increasing severity of stroke as calculated by NIHSS scores at the time of presentation. Serum GGT levels are a potential marker of ischemic stroke and its severity.

## Introduction

Gamma-glutamyl transferase (GGT) is an enzyme that belongs to the family of transferases. It catalyzes the transfer of a gamma-glutamyl group from a gamma-glutamyl peptide, such as glutathione, to a peptide or amino acid-type acceptor. Glutathione is generated during normal metabolic processes and helps in reducing oxidative stress, thereby protecting cells [1]. GGT facilitates intracellular uptake of glutathione, which is a known antioxidant. Serum GGT levels are also a useful parameter that indicates liver dysfunction and alcohol use [2,3].

Stroke is the second leading cause of death worldwide [4], causing 6.1 million deaths in 2019. Stroke is known to cause high morbidity and mortality and increases the socioeconomic burden on patients, families, and healthcare systems. The approximate stroke prevalence in different parts of India ranged from 44.29 to 559 per 100,000 persons during the past decade [5].

Ischemic stroke causes anoxic injury to the brain, which in turn releases free radicals causing glutathione to rise. This causes an increase in serum GGT levels. 

The probable mechanism that causes an increase in serum GGT level in stroke is oxidative stress. Oxidative stress is also associated with atherosclerosis. Some evidence is available which indicates a direct relation between high GGT activity and the progression of atherosclerosis [6]. There is a paucity of studies in India regarding the relationship of serum GGT with stroke. This study aimed to compare serum GGT levels in acute ischemic stroke patients with normal controls and to determine the relation of serum GGT levels with National Institute of Health Stroke Scale (NIHSS) scores.

## Materials and methods

This was a cross-sectional study conducted in a tertiary care hospital in South India from August 2023 to February 2024. The study was conducted after obtaining clearance from the Institutional Ethics Committee vide number MSRMC/EC/SP-07/08-2023. Fifty-seven cases of acute ischemic stroke who were diagnosed using a computerized tomography scan (CT scan) of the brain and admitted to the hospital were included in the study. Fifty-seven age- and gender-matched controls were included. The controls consisted of people who came for an annual health check-up at the hospital. A random venous blood sample of 5 mL was collected from the cases at the time of admission as well as from the controls at the time of visit. According to the NABL (National Accreditation Board for Testing and Calibration Laboratories)-certified laboratory in our hospitals, the reference range for serum GGT levels was 0-55 IU/L (international units per liter). Cases were classified based on NIHSS scores, areas of infarction, type 2 diabetes mellitus and hypertension status, age, and sex. Patients with an NIHSS score of 5-15 were considered moderate strokes, 16-20 were considered moderate-severe strokes, and >21 were considered severe strokes in accordance with the standard classification of NIHSS scores [7,8]. Areas of Infarction were divided in accordance with the Bamford classification, that is, anterior circulation (comprising total anterior circulation strokes and partial anterior circulation strokes), posterior circulation strokes, and lacunar strokes [9]. The serum GGT levels of the cases were compared with age- and gender-matched controls using an independent t-test. Mean serum GGT levels were compared among groups with varying NIHSS scores and different locations of infarction using the ANOVA test. Serum GGT levels were also compared based on the presence or absence of hypertension and type 2 diabetes mellitus among cases and between varying age groups and sex of the cases via an independent t-test.

Inclusion criteria

Patients aged >18 years with acute ischemic stroke diagnosed via CT scan of the brain within 24 hours of onset of symptoms were included. Age- and gender-matched controls who underwent routine health check-ups in the Internal Medicine OPD were included in this study.

Exclusion criteria

Patients with a previous history of acute ischemic stroke, liver cirrhosis, hepatitis, chronic liver disease, obstructive gallbladder disease, and cholestasis were excluded. Patients on known hepatotoxic medications such as antiepileptic drugs, anti-TB drugs, oral contraceptive pills, and methotrexate were excluded. Patients with recent consumption of alcohol (within the last four weeks) were excluded from this study. 

Sample size estimation

From a review of the literature, the mean GGT level in cases was found to be 23.3 ± 11.8 IU/L and that of the controls was 15.0 ± 5.7 IU/L (p < 0.001) [1]. In the present study, expecting similar results with 80% power and 95% confidence level, and considering the minimal detectable difference of GGT level between the two groups as 5 IU/L, our study required a minimum of 54 cases of acute ischemic stroke and 54 controls. Our study included 57 patients with acute ischemic stroke and 57 age- and gender-matched controls. Cases had a total of 43 males and 14 females, with seven patients aged 18-39 years, 23 patients aged 40-59 years, and 27 patients aged 60 years and above. Controls selected were matched to the cases and included 42 males and 15 females, with 10 patients aged 18-39 years, 17 patients aged 40-59 years, and 30 patients aged 60 years and above.

Statistical analysis

Serum GGT levels that were obtained in cases and controls were reported in terms of mean ± SD. The results were compared using the independent t-test, ANOVA test, and Pearson coefficient.

## Results

A total of 57 cases with acute ischemic stroke diagnosed via CT scan of the brain within 24 hours of onset of symptoms were divided into various groups. The values obtained in the cases were compared to those in the controls. Further, the difference in serum GGT levels among the subgroups was analyzed. The control group was comparable to the demography of the patient population (cases) with no significant difference noted between the cases and the control groups, as seen in Table 1. In Table 2, the demography of the patient population is further elaborated. 

**Table 1:** Demography of the patients in the study group

S. no.	Parameters	Mean (percent)
1.	Mean age (in years)	57.82 ± 15.37
2.	Males	43 (75.44%)
3.	Females	14 (24.56%)
4.	Type 2 diabetes mellitus	33 (57.89%)
5.	Hypertension	39 (68.42%)
6.	Anterior strokes	43 (75.44%)
7.	Posterior strokes	7 (12.28%)
8.	Lacunar strokes	7 (12.28%)

**Table 2:** Comparison of cases and controls

S. no.	Parameters	Cases	Controls
1.	Mean age (in years)	57.82 ± 15.37	54.50 ± 16.44
2.	Male:female	3.07:1	2.80:1
3.	Type 2 diabetes mellitus	33	30
4.	Hypertension	39	40

The comparison of serum GGT levels (mean ± SD) in patients diagnosed with acute ischemic stroke to the levels obtained in age- and sex-matched controls revealed a significant difference (p < 0.0001), 57 cases (43.96 ± 28.02) to 57 controls (26.14 ± 5.93) as seen in Table 3. 

**Table 3:** Serum GGT in acute ischemic stroke patients and controls GGT, gamma-glutamyl transferase. Values of serum gamma-glutamyl transferase are in IU/L (international units per liter). p-Value <0.05 is considered statistically significant.

Parameter	Number ‘N’	Mean ± SD	p-Value from unpaired t-test
Acute ischemic stroke	57	43.96 ± 28.02	<0.0001
Controls	57	26.14 ± 5.93	

Table 4 shows the comparison among three groups, which was carried out using the ANOVA method. A significant difference was noted (p < 0.00001) when we compared the serum GGT levels among different severities of stroke via the standard NIHSS score obtained at the time of presentation. The serum GGT level for 39 patients with moderate severity stroke was (5-15 points) 34.17 ± 18.39, 11 patients with moderate-severe stroke was (16-20 points) 46.63 ± 21.95, and eight patients with severe stroke was (>21 points) 84.62 ± 39.35. 

**Table 4:** Variation in serum GGT with NIHSS scores GGT, gamma-glutamyl transferase; NIHSS, National Institute of Health Stroke Scale. Values of serum gamma-glutamyl transferase are in IU/L (international units per liter). p-Value <0.05 is considered statistically significant.

Severity of stroke	Number ‘N’	Mean ± SD	p-Value from ANOVA
Moderate NIHSS 5-15	38	34.17 ± 18.39	<0.00001
Moderate-severe NIHSS 16-20	11	46.63 ± 21.95	
Severe NIHSS >21	8	84.62 ± 39.35	

**Figure 1:**
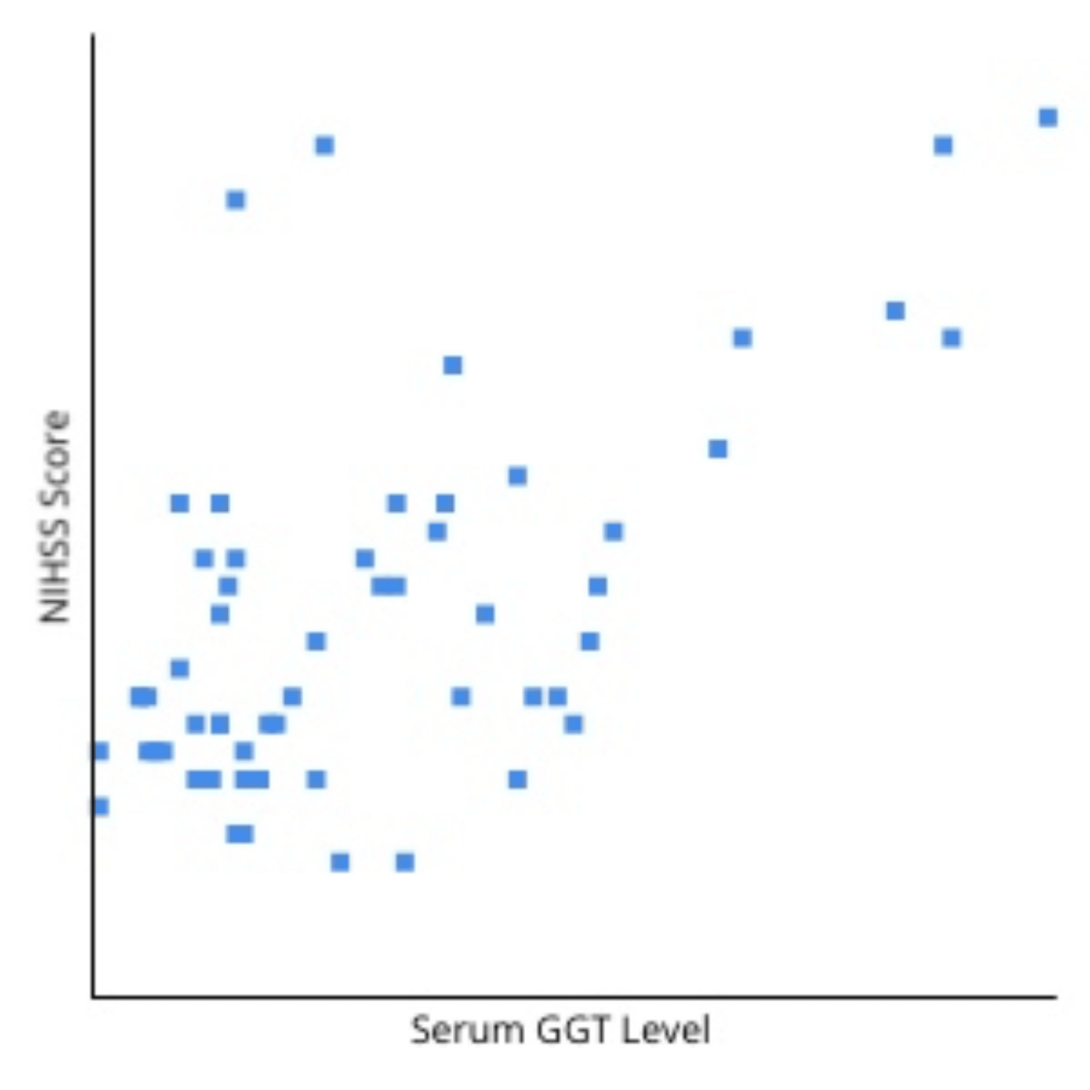
Pearson coefficient for the correlation of serum GGT with NIHSS score GGT, gamma-glutamyl transferase; NIHSS, National Institute of Health Stroke Scale. X-axis: serum GGT levels (increasing values from left to right). Y-axis: NIHSS scores (increasing severities from bottom to top). r = 0.61, p < 0.00001. p-Value <0.05 is considered statistically significant.

Figure 1 gives us the Pearson coefficient when plotting serum GGT levels against the NIHSS scores. The r-value obtained was 0.61 with p <0.00001, which showed a significant positive relation between serum GGT levels and NIHSS scores.

Table 5 compares serum GGT levels among varying locations of stroke and the age of the patient. A significant difference was not obtained (p = 0.58) when we compared the mean GGT level among 43 patients with anterior circulation stroke (45.97 ± 31.54), seven patients with posterior circulation stroke (41 ± 16.65), and seven patients with lacunar strokes (34.28 ± 7.60). A significant difference was not obtained (p = 0.34) when we compared the mean GGT level among seven patients aged 18-39 years (30 ± 17.18), 23 patients aged 40-59 years (47.95 ± 24.69), and 27 patients aged 60 years and above (44.18 ± 32.72).

**Table 5:** Variation in serum GGT with respect to area of infarction and age of the patient GGT, gamma-glutamyl transferase. Values of serum gamma-glutamyl transferase are in IU/L (international units per liter). p-Value <0.05 is considered statistically significant.

Location of stroke	Number ‘N’	Mean ± SD	p-Value from ANOVA
Anterior circulation	43	45.97 ± 31.54	0.58
Posterior circulation	7	41 ± 16.65	
Lacunar stroke	7	34.28 ± 7.60	
Age of the patients			
18-39 years	7	30 ± 17.18	0.34
40-59 years	23	47.95 ± 24.69	
60 years and above	27	44.18 ± 32.72	

Table 6 compares serum GGT levels of patients based on type 2 diabetes mellitus and hypertension status of the cases and gender of the cases. A significant difference was not obtained (p = 0.28) when we compared the mean GGT level among 33 patients with type 2 diabetes mellitus (45 ± 29.39) to 24 patients without type 2 diabetes mellitus (37.54 ± 20.28).

A significant difference was not obtained (p = 0.71) when we compared the mean GGT level among 39 patients with hypertension (42.74 ± 25.90) to 18 patients without hypertension (39.94 ± 27.10).

A significant difference was not obtained (p = 0.07) when we compared the mean GGT level among 43 male patients (45.37 ± 27.82) to 14 female patients (31.07 ± 15.93).

**Table 6:** Variation in serum GGT levels with type 2 diabetes mellitus, hypertension, and sex of the patient GGT, gamma-glutamyl transferase. Values of serum gamma-glutamyl transferase are in IU/L (international units per liter). p-Value <0.05 is considered statistically significant.

Parameter	Number ‘N’	Mean ± SD	p-Value from unpaired t-test
With type 2 diabetes mellitus	33	45 ± 29.39	0.28
Without type 2 diabetes mellitus	24	37.54 ± 20.28	
With hypertension	39	42.74 ± 25.90	0.71
Without hypertension	18	39.94 ± 27.10	
Males	43	45.37 ± 27.82	0.07
Females	14	31.07 ± 15.93	

## Discussion

The study dealt with the level of serum GGT and acute ischemic stroke among patients admitted to a tertiary care hospital in South India. Our study shows that serum GGT levels were significantly elevated in patients with acute ischemic stroke as compared to control subjects. This result was concordant with the result of the study conducted by Gurbuzer N et al. [1] as well as the studies conducted by Ismail et al. [10] and Singh LK et al. [11], which revealed a higher serum GGT level in cases of acute ischemic stroke in comparison to control subjects.

The results of our study indicated that serum GGT levels were statistically significant with the severity of stroke as determined by the NIHSS score. A statistically significant relation was observed when we compared serum GGT levels among increasing severities of stroke, thereby establishing the fact that serum GGT levels increase with increasing severities of stroke. The serum GGT levels and NIHSS scores can give us important information about the patient's status. These findings were concordant with the study conducted by Yao T et al., which demonstrated that partial correlation analysis showed a significant association of serum GGT level with NIHSS score at admission after adjustment for age and gender (r = 0.150, p = 0.001) [12].

Our study showed no significant association in serum GGT levels with various locations of stroke, suggesting no role in differentiating between various territories involved solely on serum GGT levels. The serum GGT levels were not significantly associated when comparing cases with hypertension or type 2 diabetes mellitus to non-hypertensive or non-diabetic cases, respectively. This result suggests that the presence of type 2 diabetes mellitus or hypertension does not play a role in elevating serum GGT levels in patients [13]. This result was different from the one obtained in the study conducted by Gurbuzer N et al. [1]. Our study also differs from the study conducted by Ismail et al. [10] in that serum GGT levels were not significantly different when comparing patients by age or sex. However, this result from our study supports a similar observation to the one in the study conducted by Kalirawna TR et al. [14] in which serum GGT levels were significantly elevated in patients with acute ischemic stroke irrespective of the patient's age or sex, which suggests that molecular mechanisms that play a role in the elevation of serum GGT levels in acute ischemic stroke are similar for patients of varying age groups and sex.

These results further establish the fact that the increase in serum GGT levels in stroke is due to molecular mechanisms caused by acute anoxic injury to the brain parenchyma.

Similar studies have established a relation between oxidative stress and serum GGT level, along with molecular mechanisms that play a role in situations of subclinical inflammation often seen in cardiovascular disease and stroke [15,16].

Some studies have also shown a positive relation between higher levels of serum GGT and their association with silent brain infarctions in a healthy population [17]. Studies have also shown that serum GGT levels can serve as an independent marker for predicting cardiovascular and all-cause mortality and may be independent of alcohol intake [18-21].

Further studies will help establish the role of serum GGT levels in the prognosis of stroke and its outcomes [22,23]. Prospective cohort studies will help us understand the role of NIHSS scores and serum GGT levels in predicting mortality in patients with acute ischemic stroke. 

Serum GGT levels, in conjunction with NIHSS scores, can prove useful in low-resource settings such as in rural areas, where the facility of CT scans may not be readily available, as they can be used as a soft marker suggestive of stroke in patients. 

As serum GGT and glutathione are closely related, this may also help establish the role of antioxidants in the management and outcomes of stroke [24].

Our study did have its limitations. First, this study was conducted in a single center with a limited sample size. Second, serum GGT levels of the cases were obtained at the time of admission and studied. Further prospective multicentric studies conducted with a larger sample size should be conducted to determine the role played by serum GGT in cases of acute ischemic stroke and its relation with NIHSS scores. Studying the serial trend of serum GGT will also shed light on its role as a prognostic marker with respect to the outcomes of stroke.

## Conclusions

Serum GGT levels are significantly elevated in patients with acute ischemic stroke. There is a statistically significant difference when serum GGT is compared among patients with varying NIHSS scores. Elevation in serum GGT levels is greater in severe strokes as compared to moderately severe strokes. Serum GGT levels are a potential marker of stroke and its severity. Further studies with a larger sample size are required to establish the use of serum GGT levels as a surrogate marker for the detection and estimation of the severity of stroke. Serum GGT may also play a key role in predicting the outcome of a stroke.
